# Integrated Whole-Genome Resequencing and Transcriptomic Analyses to Reveal Breed-Specific Selection Signatures of Cardiac-Related Genes in Wuzhishan Pigs

**DOI:** 10.3390/biology15141214

**Published:** 2026-07-22

**Authors:** Yuwei Ren, Feng Wang, Xinli Zheng, Yan Zhang, Ruiyi Lin, Xuyang Lu, Pengpeng Zhang, Cencen Li, Linlin Chen, Zhe Chao

**Affiliations:** 1 Key Laboratory of Tropical Animal Breeding and Disease Research, Institute of Animal Science and Veterinary Medicine, Hainan Academy of Agricultural Sciences, Haikou 571100, China; renyuwei@hnaas.org.cn (Y.R.);; 2 College of Animal Sciences, Fujian Agriculture and Forestry University, Fuzhou 350002, China; linruiyi@fafu.edu.cn; 3 Department of Biotechnology, College of Life Sciences, Xinyang Normal University, Xinyang 464000, China

**Keywords:** whole-genome resequencing, high and low nucleotide diversity regions, cardiac-related genes, cardiac muscle contraction, Wuzhishan pigs

## Abstract

The WZS minipig, native to the Hainan province of China, is characterized by excellent meat palatability. In addition, this breed represents a promising animal model for human disease research, especially for cardiac diseases. Whole-genome resequencing is a powerful approach for guiding breeding decisions. By identifying mutations and conserved regions linked to environmental adaptability and physiological fitness, this method supports both the genetic improvement in pig breeds and their application as animal models for human disease research. However, the genomic locations of high and low nucleotide diversity regions harboring cardiac-related genes in the WZS genome remain unclear. In this study, cardiac-related genes were selected based on whole-genome resequencing data previously released by our research team, including 147 samples from five breeds (PRJCA054653), and detected RNA expression patterns of heart tissues across five growing periods. Between-breed analysis indicated that WZS pigs exhibited the highest nucleotide diversity among the five breeds and underwent a recently diverged lineage. A non-redundant 236-gene set was identified from the union of three gene subsets, of which the functional genes enriched in KEGG (67) and GO terms (192) were primarily implicated in cardiac muscle structure and energy metabolism, supplemented by additional genes selected based on high expression levels (52) and DEGs (44). Specifically, the DEGs of *ACTC1*, *TNNC1*, *TNNT2*, *COX4I1*, and *MYH7* were highly expressed in the heart and enriched in the cardiac contraction pathway. The findings provide candidate genes for breeding and support the future use of WZS pigs as an improved animal model for human cardiac research.

## Simple Summary

The Wuzhishan (WZS) minipig is a famous pig in the Hainan province of China, known for its excellent meat palatability, and taken as a promising animal model for human cardiac disease research. Whole-genome resequencing enables the detection of mutational and conserved regions for breeding applications, while well-characterized pig breeds can facilitate the development of animal models. Here, whole-genome resequencing was combined with cardiac RNA-seq data across five developmental stages to screen for cardiac-related genes. A total of 236 non-redundant genes were selected from the union gene set of three pipelines. From the gene set, 67 and 192 genes were respectively enriched in the top 20 KEGG pathways and GO terms, 52 genes exhibited high expression, and 44 genes were differentially expressed between any two of the five developmental stages. These genes may serve as valuable targets for developing WZS pigs as animal models of human cardiac diseases and for genetic breeding to improve cardiac function.

## 1. Introduction

The Wuzhishan (WZS) pig is a minipig native to Hainan Province, China, characterized by a black head and back, a white belly, and a distinct white triangular patch on the forehead. Known for their flavorful meat and resilience to the hot, humid subtropical climate, WZS pigs also serve as valuable models for biomedical research [1], and are predicted to hold promise for applications in human disease investigation.

Animal models have long been instrumental in developing treatments and vaccines for human diseases, ranging from small rodents to large mammals. Pigs are increasingly favored due to their physiological, immunological, and genetic similarities to humans [2]. In cardiovascular research, porcine models have been pivotal in heart valve and vascular replacement studies [3] and coronary stent development for preclinical trials [4]. Indeed, porcine heart models have been investigated for over 50 years [5]. In 1968, when aortic valve xenografts were first introduced, less than 50% of transplanted valves remained functional beyond one year [6]. Recent years have witnessed the emergence of pig-to-human cardiac xenotransplants, exemplified by the procedures at the University of Maryland [7,8]. In order to promote the development of porcine models for myocardial research, it is urgent to provide an excellent breed, and analysis of whole-genome sequence data is an effective approach to achieve this objective as it could precisely identify the key genes governing important traits [9].

Cardiac contraction and relaxation rely on the intricate crosstalk between the cytoskeleton and mitochondria, which govern structural integrity and energy supply, respectively [10]. The cytoskeletal network includes thin and thick filaments and an intracytoplasmic filamentous system [11], while mitochondria generate ATP to sustain cellular function. Cardiac contraction is regulated by troponins, and inhibition of troponins induces muscle relaxation. Upon activation, actin in the thin filament binds to myosin in the thick filament, forming cross-bridges that drive contraction [12]. Microtubules further interconnect cytoplasmic components, while intercalated discs facilitate electrical signaling between cardiomyocytes [13]. These intracellular and extracellular structures collectively maintain cardiac function.

Functional genes have undergone mutation and fixation throughout evolution. The genomic regions with low nucleotide diversity regions developed into conserved genes, and mutated regions exhibited high nucleotide diversity regions [14]. Mutation enhanced biodiversity and contributed to the adaptive responses to the rapidly changing environments and defend against pathogens; while regions with low nucleotide diversity can be important for regulating the expression of genes contributing to vital cellular processes, such as cell cycle, DNA repair, and energy metabolism [15]. Given that high nucleotide diversity is generally associated with adaptive potential in response to environmental pressures [15], the high nucleotide diversity identified in the pig heart may confer adaptive advantages under drastic physiological changes. Conversely, regions with low nucleotide diversity might be crucial for maintaining basal cardiac functions, potentially by regulating essential gene expression. However, whether cardiac-related genes possess conserved and mutated regions, and how these features contribute to cardiac functions, remain to be determined. WZS pigs exhibit distinct morphology, small body size, and unique coat coloration, and are geographically isolated on Hainan Island, which was beneficial for driving genetic divergence, adaptive evolution, and the development of novel phenotypes beneficial for human applications [16,17]. This study is aimed at investigating the breed-specific selection signatures in cardiac tissue genes of WZS pig by integrating whole-genome resequencing and transcriptomic analyses, and establishing a fundamental basis for the development of porcine cardiac models for human disease research.

## 2. Materials and Methods

### 2.1. Datasets for Whole-Genomic Resequencing

The sequencing data used in this study were derived from 147 samples across five pig breeds, previously released by our research team (PRJCA054653) [18]: 30 Wuzhishan (WZS, 4 months old) and 30 Tunchang (TC, 6 months old) pigs from Hainan Province; 29 Yuxi (YX, 8 months old) pigs from western Henan Province; and 30 Large White (LW, 2 months old) and 28 Duroc pigs (8 months old) from Guangdong Province (Figure S1).

### 2.2. Variant Calling

Quality control of raw sequencing data was performed using FastQC v0.11.9 to evaluate base quality, GC content, and adapter contamination. Low-quality reads and adapter sequences were removed using Trimmomatic (v0.39) with default parameters. Clean data were aligned to the WZS pig reference GCA_057782405.1 (National Center for Biotechnology Information) using Burrows–Wheeler Aligner (BWA-MEM v0.7.17). The aligned reads were sorted using SAMtools (v1.20) [19], and duplicate reads were removed using the MarkDuplicates module in GATK (v4.2.2.0). Single-nucleotide polymorphisms (SNPs) and small insertions/deletions (indels) were called using the HaplotypeCaller module in GATK to generate individual gVCF files [20]. Only variants with a sequencing depth >6× were retained for analysis. Combined gVCF files were processed with GenotypeGVCFs to obtain breed-level SNPs and indels. SNPs were filtered using SelectVariants with the parameter “–select–type–to–include SNP” and further refined with VariantFiltering using the criteria: QD < 2.0, MQ < 40.0, FS > 60.0, SOR > 3.0, MQRankSum < −12.5, and ReadPosRankSum < −8.0, and further filtered missing genotypes >10% and MAF > 0.05 using PLINK (v2.0) [21]. SNPs were annotated using SnpEff [22] based on the WZS pig reference genome.

### 2.3. Between-Breed Genetic Difference

High-quality SNPs were used to assess genetic diversity and between-breed differentiation based on the fixation index (FST), nucleotide diversity (π), π ratio, and Tajima’s D. Nucleotide diversity (π) was calculated using 100 Kb non-overlapping windows with VCFtools (v0.1.17) [23]. Between-breed differentiation was evaluated using Weir and Cockerham’s *F_ST_* estimator [24] with a 100 Kb window via VCFtools v0.1.17. Linkage disequilibrium (LD) decay was estimated using the squared correlation coefficient (r^2^) at a maximum distance of 300 kb with PopLDdecay (v3.41) [25].

### 2.4. Between-Breed Genetic Analysis

The identity-by-state (IBS) genetic distance matrix was calculated using PLINK (v2.0) [26], and a phylogenetic tree was constructed using the neighbor-joining (NJ) algorithm in Phylip [27]. Principal component analysis (PCA) was performed using the R package FactoMineR (version 2.12) to infer breeds’ clustering patterns.

Historical effective population size (Ne) was estimated using the Pairwise Sequentially Markovian Coalescent (PSMC) model [28]. The mutation rate was set at 3.6 × 10^−9^ per nucleotide per generation, with a generation time of three years, allowing estimation of Ne from 1 Kya to 10 Mya [29]. Population structure was analyzed using ADMIXTURE (v1.3.0). Genomic inbreeding coefficients based on runs of homozygosity (FROH) were calculated with PLINK (version 2.0). The relative divergence times among pig breeds were estimated using BEAST2 (v2.7.8), and the fossil time of Sus scrofa is 62 Mya (http://www.timetree.org/).

### 2.5. The High and Low Nucleotide Diversity Regions Analysis

Windows within the top 5% of the genome-wide FST and π ratio distributions were used to identify genomic regions undergoing selection. This threshold value was chosen as a balance between sensitivity and specificity, as stricter cutoffs (e.g., 1%) would substantially reduce the number of candidate genes, while more lenient cutoffs (e.g., 10%) would increase false positives. The cutoff of the top 5% FST and π ratio is commonly used in livestock selection signature analyses [30,31].

The statistical evidence of nucleotide diversity regions was analyzed by π proportion difference. First, the WZS π value of the window corresponding to the minimum value of the top 5% FST (WZS pigs vs. the other four pig breeds) was selected as the threshold value. Subsequently, π values exceeding this threshold were retained for all five pig breeds, and the proportion differences (along with its 95% confidence interval) between WZS and each of the other breeds were calculated, defined as π_WZS minus π_other.

The gene set was obtained by intersecting the genomic intervals of outlier windows with the genomic intervals of annotated genes from the reference genome using BEDTools (version 2.31.1) with parameter -f 1.0. The genes of the top 5% FST and π ratio regions were used for KEGG enrichment, and the genes in the top 20 pathways were reserved for further analysis.

### 2.6. Transcriptome Data Analysis

Fifteen WZS pigs, including three individuals (2 sows and 1 boar) for each five growth periods (3-day-old, 2-month-old, 4-month-old, 8-month-old, 12-month-old), were purchased from the Nongkenhongmu Agricultural Development Company (Qiongzhong, Hainan, China). All pigs were weaned at 2 months of age. The 15 piglets were sampled from 5 sows, with 3 pigs per time point (one sow per time point). The 15 pigs were farmed with ad libitum food and water for one week before slaughter. After 24 h of feed withdrawal, the pigs were stunned by electric shock and exsanguinated, flushed, and split in a state of unconsciousness. The heart tissues were collected, frozen directly in liquid nitrogen and stored at −80 °C before RNA extraction. Total RNA was extracted from tissues using an RNA extraction kit (Qiagen, Hilden, Germany, Cat. No./ID: 74104), and RNA-seq was performed on the prepared libraries using the Illumina NovaSeq 6000 System by the Novogene Corporation (Beijing, China) to obtain ~6 GB data for each sample. Raw data were filtered using fastp (v1.0.1) to remove adapters and low-quality reads, clean reads were mapped to the WZS pig reference [18] GCA_057782405.1 (National Center for Biotechnology Information) using HISAT2 (v2.2.1) with default parameters, and TPM was calculated by RNAnorm (v2.0.1) using gene count and gene length. Genes with TPM ≥ 1 detected in all samples were considered as expressed genes. Differentially expressed genes (DEGs) were analyzed using R package DESeq2 (v1.52.0) according to the requirements of |log_2_ ^(foldchange)^| ≥ 1, and adjusted by *p* adjust ≤ 0.05 [32]. DEGs were used for KEGG enrichment analysis according to the statistical threshold *p* adjust ≤ 0.05.

### 2.7. The Selection of Cardiac-Related Genes

The initial gene set was obtained from the top 5% FST and π ratio regions (π ratio: WZS pigs vs. the other four pigs, and the other four pigs vs. WZS pigs). The cardiac-related genes were selected using the following criteria:

Firstly, KEGG and GO enrichments were performed on the initial gene set, and retained the genes assigned to the top 20 KEGG pathways and the top 20 GO terms; secondly, among the genes identified in the initial gene set, those with high expression (i.e., top 5%) in heart tissues across the five growing periods were retained; thirdly, DEGs were identified between any two of the five examined growth periods within the initial gene set, and those with expression levels in the top 5% were retained; and finally, the genes identified by these three selection procedures were merged, and duplicate genes were removed to obtain the final cardiac-related gene set (Figure S2).

### 2.8. Collinearity Analysis

MCScanX (v1.0.0) was used to identify the collinearity blocks between the WZS and Duroc pig, and the WZS pig and homo sapiens with default parameters. Genome annotation (GFF3) and protein sequences were downloaded from NCBI RefSeq under assembly accession GCF_000003025.6 (Sus scrofa 11.1), GCF_000001405.40 (homo sapiens), and figshare (https://figshare.com/s/57033524d6fcec0afdc6, WZS pigs, accessed on 19 July 2026).

## 3. Results

### 3.1. Overview of Between-Breed Variations

To identify selection signatures unique to WZS pigs, WZS pigs were compared against the other four pig breeds (Tunchang (TC) pigs, Yuxi (YX) pigs, Large White (LW) pigs, and Duroc pigs). SNPs were called from the data previously released by our research team (PRJCA054653) [18]. A total of 22,996,935 SNPs were identified in the WZS pig DNA samples compared to the other four pig breeds (Table S1). Among the five breeds, WZS pigs exhibited the highest nucleotide diversity (Table S2). Most SNPs were located in intergenic regions (65.19%), followed by intronic regions (27.64%), while coding sequences (CDS) accounted for 0.84% in WZS pigs (Figure S3). This distribution was similar to that observed in YX, Duroc, and LW pigs.

### 3.2. Between-Breed Genetic Differences

**Figure 1:**
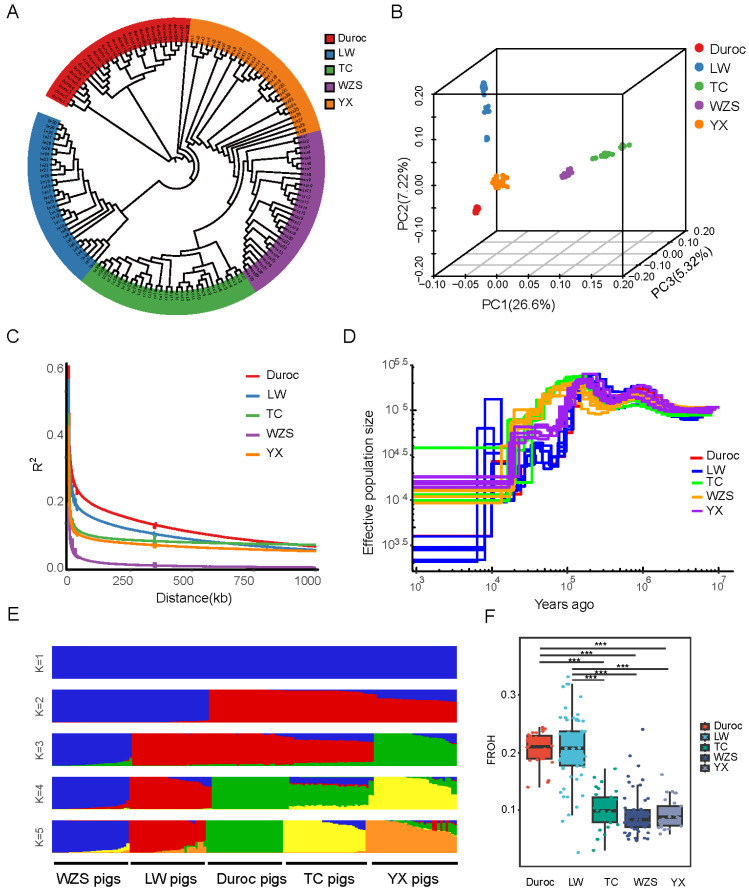
The genetic analysis of five pig breeds. (**A**) The phylogenetic tree was constructed by SNPs of 147 samples from five pig breeds using PLINK based on neighbor-joining (NJ) algorithm. (**B**) Principal component analysis (PCA) was performed based on genome-wide SNP data from five pig breeds, with dots colored by pig breeds. (**C**) Linkage disequilibrium (LD) decay was estimated using the coefficient (r^2^) with a maximum distance of 300 kb. (**D**) Effective population size (Ne) of five pig breeds was analyzed by PSMC. The horizontal axis represents the time periods, and the vertical axis represents the Ne value. (**E**) Admixture bar plots of five pig breeds from 147 individuals were analyzed for K = 1 to K = 5. Each vertical bar represents an individual pig, and the colored segments within each bar represent the estimated proportion of ancestry from K inferred ancestral populations. Results are shown for K = 1 through K = 5 (from top to bottom). At K = 5, the five breeds are clearly distinguished by five distinct ancestral components. Individuals are grouped by pig breeds, and pig breed names are labeled at the bottom: WZS pigs, LW pigs, Duroc pigs, TC pigs and YX pigs. (**F**) Boxplot of genomic inbreeding coefficients (FROH) across five pig breeds. FROH was calculated as the total length of ROH divided by the total autosomal genome length. Within each box, the center line represents the median FROH, the box bounds represent the interquartile range (IQR, 25th to 75th percentile), and the whiskers extend to 1.5 × IQR beyond the box. Points beyond the whiskers indicate outliers. The *x*-axis represents pig breeds, and the *y*-axis represents FROH values. Significant differences between breeds are indicated by asterisks (*** *p* < 0.001) above the boxes.

To evaluate whether the five pig breeds could be genetically distinguished from each other, a phylogenetic tree was constructed based on SNPs from all 147 samples using the neighbor-joining (NJ) algorithm implemented in PLINK. The phylogenetic tree showed that the five pig breeds were clearly separated into five distinct clusters (Figure 1A). WZS and TC, as well as WZS and YX pigs, were genetically closer to each other. In contrast, LW and Duroc pigs were positioned at opposite ends of the tree, showing a greater genetic distance from WZS pigs. Notably, despite all being Chinese native pig breeds, WZS, TC, and YX pigs formed completely separate branches in the tree and showed no overlap in PCA analysis (Figure 1B).

Between-breed differences within and between pigs were assessed using FST and π. WZS pigs presented the highest nucleotide diversity (π) of 0.0019 ± 0.009, with 95% of the 100 Kb sliding windows falling between 0.0011 and 0.0026, while Duroc pigs showed the lowest π of 0.0011 ± 0.008, with 95% of the 100 Kb sliding windows falling between 0.0004 and 0.0017 among the five pig breeds (Table S3). Tajima’s D analysis indicated that WZS pigs experienced balancing selection and population contraction, as most genomic regions exhibited values greater than zero (Figure S4). The lowest Pairwise FST comparisons were observed between WZS and TC pigs (FST = 0.1313 ± 0.0776), with 95% of the 100 Kb sliding windows falling between 0.0776 and 0.1686, indicating a closer genetic relationship, whereas the highest FST was between WZS and Duroc pigs (FST = 0.4463 ± 0.1792), with 95% of the 100 Kb sliding windows falling between 0.3145 and 0.5632 (Table S3), suggesting relatively small genetic divergence between WZS and TC pigs, and substantial genetic divergence between WZS and Duroc pigs. Linkage disequilibrium (LD) decay analysis revealed that Duroc pigs had the highest LD levels, while WZS pigs exhibited the lowest (Figure 1C). The LD decay rate in WZS pigs was faster than the other four pig breeds, indicating a larger historical effective population size and higher recombination rate in WZS pigs. Based on the relative divergence times estimated by BEAST2 (v2.7.8), WZS and TC pigs appear to have diverged later than the other three pig breeds (Duroc, LW and YX pigs) (Figure S5).

### 3.3. Genomic Nucleotide Diversity Regions

The analysis of Ne values showed a general increase in Ne across the five pig breeds approximately 10^5^ years ago, followed by a decline. Notably, the decline in Ne was more gradual in WZS and TC pigs compared to the other three pig breeds (Figure 1D). The genetic ancestry of the five pig breeds was estimated using ADMIXTURE at K = 1 to K = 5 (Figure 1E). At K = 2, the individuals were partitioned into two ancestral clusters; at K = 3 and K = 4, the number of ancestral components increased progressively, with breeds gradually separating from one another. At K = 5, the five pig breeds were completely distinguished by five distinct ancestral components, with each breed predominantly assigned to a single unique component. Overall, Duroc pigs exhibited the highest genomic inbreeding levels, with a median FROH of 0.207 (range: 0.178–0.235) (Table S4). In contrast, WZS pigs showed the lowest FROH of 0.027 (range: 0.052–0.106), indicating a more genetically diverse background of WZS pigs compared to the other four pig breeds. Furthermore, TC, YX and WZS pigs showed lower FROH distributions than those of Duroc and LW pigs, and statistical comparisons using the Wilcoxon test revealed that FROH differed significantly between Duroc pigs and TC, YX, and WZS pigs, and between LW pigs and TC, YX, and WZS pigs (*p* < 0.001), respectively (Table S5) (Figure 1F). Integrating these findings, the WZS pig genome exhibited more nucleotide diversity than the other four pig breeds in this study.

**Figure 2:**
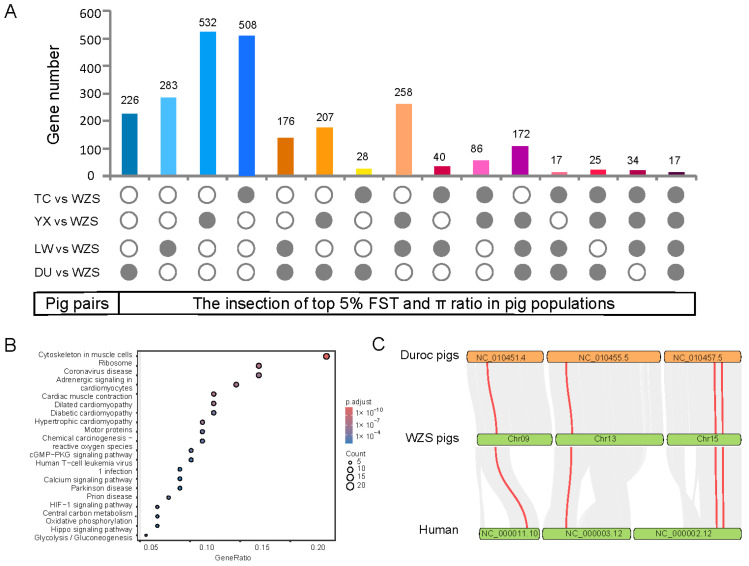
The selection of cardiac-related genes with low nucleotide diversity regions. (**A**) Intersection of top 5% FST and π ratio genes between WZS and the other four pig breeds. The upper panel shows the number of genes identified by the intersection of the top 5% FST and top 5% π ratio for each breed comparison. The UpSet plot displays all 15 intersection combinations among the four groups (the top 5% FST and top 5% π ratio (the other four pig breeds vs. WZS pigs)), ranging from single-group to four-group intersections. In the lower panel, the gray circles in columns one to four represent the gene intersections between the WZS pig and each of the other four pig breeds, respectively; two gray circles in columns five to ten represent the gene intersections between WZS pigs and any two of the other four pig breeds; three gray circles in the columns 11 to 14 represent the gene intersections between WZS pigs and any three of the other four pig breeds; and four gray circles in column 15 represent the gene intersections between WZS pigs and the other four pig breeds. Numbers on the top of each bar indicate the gene number in each intersection set. (**B**) KEGG pathway enrichment analysis of the 67 candidate genes (top 20 pathways). The *x*-axis (gene ratio) represents the number of genes annotated to a pathway divided by the total number of genes in that pathway. Dot size indicates the number of enriched genes; dot color indicates the adjusted *p*-value. (**C**) The collinearity analysis was performed between WZS and Duroc pigs, and between WZS pig and humans, using MCScanX with default parameter. The e-value threshold for BLAST (version 2.14.0) homology detection was set to 1 × 10^−5^. Genes of *ATM*, *TNNC1*, *COL3A1*, and *BMPR2* are posited on chromosomes 9, 13, 15 and 15, respectively.

To identify breed-specific selection signals, the intersection of the top 5% FST genes and top 5% π ratio genes for each of the four pig breeds was compared against WZS pigs. The intersection yielded 226 genes for Duroc vs. WZS (Table S6), 283 genes for LW vs. WZS (Table S7), 532 genes for YX vs. WZS (Table S8), and 508 genes for TC vs. WZS (Table S9). These breed-specific genes represented low nucleotide diversity regions (Figure 2A, Table S10). Furthermore, the statistical evidence of nucleotide diversity regions was analyzed by π proportion differences. The proportion of nucleotide diversity regions varied substantially across breeds. As the WZS π value 0.00206805 was the window corresponding to the minimum value of top 5% FST (WZS pigs vs. the other four pig breeds), this π value was taken as the threshold to screen out the high nucleotide diversity regions of the five pig breeds. WZS exhibited the highest nucleotide diversity regions proportion (π%: 41.5%, 95% CI: 41.3–41.7%) (Tables S11 and S12), whereas Duroc pigs showed the lowest (π%: 15.7%, 95% CI: 15.6–15.9%) (Table S12). The proportion difference in the π value between WZS and Duroc pigs reached 25.7% (95% CI: 25.5–26.0%), representing an approximately 2.6-fold nucleotide diversity region in WZS relative to Duroc pigs, which was higher than the proportion differences in the π values between WZS and the other four pig breeds. This substantial disparity indicated that WZS maintains a markedly higher level of genomic polymorphism compared to the other four pig breeds.

### 3.4. Cardiac-Related Gene Selection by Intersection of Selection Signatures and Transcriptome Analysis

To select cardiac-related genes in WZS pigs, an initial gene set was first established by genome-wide selection signature scanning between WZS and four other pig breeds, which was the union of the top 5% FST and top 5% π ratio regions (WZS vs. the other four pig breeds, the other four pig breeds vs. WZS pigs) with TPM > 1, which yielded 1095 genes (Table S13). From this set, three pipelines were applied to select cardiac-related genes: first, KEGG and GO enrichment analyses were performed to retain genes assigned to the top 20 pathways and GO terms, respectively; second, RNA levels in heart tissue were examined, and those within the top 5% percentile were retained; and third, identification of the top 5% differentially expressed genes (DEGs) across five developmental stages. Finally, the genes retained from the three pipelines were combined, and duplicates were removed, yielding a non-redundant set of 236 genes.

The top 20 KEGG pathways, derived from 67 non-redundant genes, included those significantly enriched in cardiac muscle contraction, cardiomyopathy, calcium signaling, gluconeogenesis, ribosome, and cytoskeletal organization in muscle cells (Figure 2B, Table S14). The relative proportion of KEGG-annotated genes (67/1095) appeared low, potentially due to the limited coverage of KEGG pathway annotations for pigs. To obtain broader functional coverage, Gene Ontology (GO) enrichment analysis was additionally performed; the top 20 enriched GO terms were concerned with the regulation of contractile muscle fiber and mitochondrial activity (Figure S6, Table S15).

The intersection of genes within the top 5% FST and π ratio (the other four pig breeds vs. WZS pigs) and those in the top 20 KEGG pathways yielded 15 conserved genes. Among the 15 genes, four (collagen type III alpha 1 chain (*COL3A1*), troponin C1 (*TNNC1*), ataxia telangiectasia-mutated (*ATM*), and bone morphogenetic protein receptor type 2 (*BMPR2*)) harbored low nucleotide diversity regions and showed high homology and chromosomal synteny with humans (Table S16), suggesting their potentially important functions in heart tissue. Additionally, *TNNC1* and *COL3A1* contained both high and low nucleotide diversity regions, while *COL3A1* and *BMPR2* were both located on chromosome 15 (Figure 2C and Table S17).

**Figure 3:**
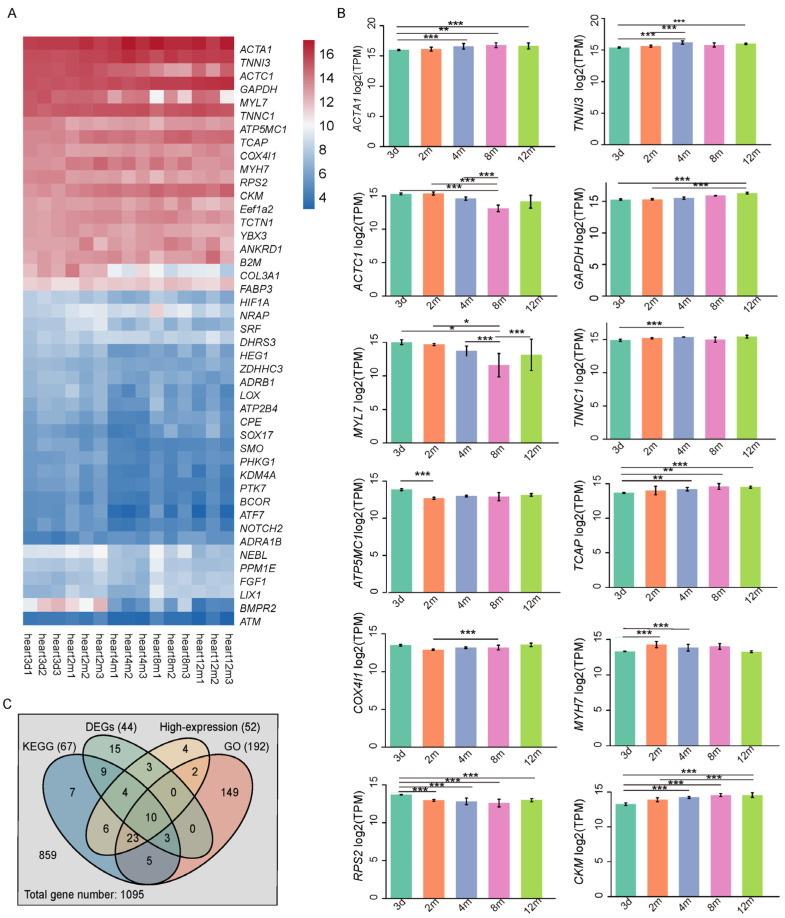
Expression analysis of selected cardiac-related genes. (**A**) The expression (log_2_^TPM^) of 44 cardiac-related genes in heart tissue from five growing periods. The DEGs were analyzed using DESeq2 according to the requirements of|log_2_^FC^| ≥ 1, and adjusted by *p* ≤ 0.05. (**B**) The expression (log_2_^TPM^) of the top 12 DEGs with high expression levels in heart tissues. T−shaped error bars represent SD across 3 values per group. Significance levels are indicated as follows: * *p* ≤ 0.05, ** *p* ≤ 0.01 and *** *p* ≤ 0.001. (**C**) Venn diagram showed the overlap among the four cardiac-related gene sets. There were 67 genes of KEGG enrichment, 192 genes of GO enrichment, 52 high-expressed genes and 44 DEGs in heart tissue from five growing periods.

Among the 1095 genes identified from the genomic selection scan, 52 showed consistently high expression across all five growing stages (the top 5% of the initial gene set, 52/1095). In parallel, a total of 880 DEGs (|log_2_^FC^| ≥ 1, *p* adjust ≤ 0.05) were identified in heart tissue across five growing stages, and 44 DEGs were selected from the top 5% of total DEGs (44/880) (Figure 3A, Table S18). DEGs (*TNNI3*, *TNNC1*, *MYH7*, *ACTA1*, and *TCAP*) were gradually increased during the growing period, whereas *MYL7* was the highest at the 3 days old age, and then began presenting a downward trend. The glycolysis-related gene *GAPDH* increased from 3-day-old to 12-month-old age, whereas DEGs of ATP synthase membrane subunit c locus 1 (*ATP5MC1*) and cytochrome c oxidase subunit 4 isoform 1 (*COX4I1*) decreased with increasing age (Figure 3B, Table S19).

In summary, the union of three subsets, including functional enrichment genes implicated in cardiac muscle structure and energy metabolism (KEGG-enriched genes (67), GO-enriched genes (192)), high-expression genes (52), and DEGs (44), yielded a non-redundant 236-gene set (Figure 3C, Table S20).

## 4. Discussion

### 4.1. Selection Signatures of WZS Pigs

Between-breed genetic analysis between WZS pigs and the other four pig breeds reflected the selection signatures of WZS pigs, which are determined by nucleotide diversity, genetic selection, linkage disequilibrium (LD), and effective population size (Ne). LD analysis revealed that the rate of decay in WZS pigs was faster than in the other four pig breeds, and the FROH values of WZS pigs were lower than those of the other four pig breeds, indicating the historical effective population size of WZS pigs was large, the recombination rate was high, and the inbreeding rate was low. The Ne of WZS pigs has remained larger than the other four pig breeds since ~10^5^ years ago, suggesting WZS pigs have a large effective population size at that period. Ne analysis showed that WZS pigs have maintained a large Ne around 1 Mya, and decreased from that period. PCA and ADMIXTURE analysis revealed that WZS pigs form a single homogeneous breed without clear sub-structuring, suggesting that population stratification is not a major contributor to the high nucleotide diversity. Taken together, the higher nucleotide diversity in WZS pigs is most likely explained by a relatively large effective population size, rather than recent introgression. This result contradicted previous studies that WZS pigs diverged earliest from mainland pig breeds, even before Duroc and LW pigs [33]. The majorly possible reasons might be methodological differences, as a previous study constructed a phylogenetic tree using single-copy orthologous genes from different species, while this study constructed a phylogenetic tree using SNPs based on whole-genome resequencing data from five pig breeds.

The nucleotide diversity regions analysis between five pig breeds indicated that WZS and Duroc pigs harbored the highest proportion of nucleotide diversity difference (25.7%). This substantial difference in nucleotide diversity regions proportions is unlikely to be caused by stochastic variation, given the large number of windows analyzed (*n* = 248,426 per breed). Instead, this disparity likely reflects basic differences in population demography and selection history. The lower nucleotide diversity regions proportion in Duroc pigs is consistent with the effects of intensive artificial selection or population bottlenecks, which reduces genome-wide nucleotide diversity. In contrast, the high nucleotide diversity regions proportion in WZS suggests either a larger effective population size (Ne) or the action of balanced selection maintaining multiple allele mutation sites. This observation aligns with the lower number of conserved regions identified in WZS, as regions with high polymorphism are less likely to be evolutionarily constrained.

### 4.2. Screening of Genes Related to Cardiomyocyte Structure and Energy Metabolism

In this study, KEGG and GO enrichment and transcriptome analyses were performed to select genes in heart tissue. In detail, a total of 1095 expressed genes were selected from the intersection of the top 5% FST and π ratio regions. This initial gene set was used to select 236 redundant cardiac-related genes by three pipelines, including functional enrichment genes (67 genes from the top 20 KEGG pathways and 192 genes from the top GO terms), high-expression genes in the heart across five growing periods (52 genes), and the top expression-lever DEGs (44 DEGs).

Genes significantly enriched in KEGG pathways were related to the cytoskeleton in muscle cells, cardiac muscle contraction, calcium signaling, cardiomyopathy, and gluconeogenesis, which suggested that the selected genes were primarily associated with cardiac muscle structural integrity and energy metabolism. Myosin molecules constitute the fundamental structural component of cardiac muscle [11]. Cardiac contraction requires substantial energetic demands from cardiac muscle [34]. Heart tissue is enriched with mitochondria, which are multifunctional organelles involved in diverse processes such as energy production and the biosynthesis of iron–sulfur clusters. Mitochondrial Ca^2+^ was correlated with intracellular ATP levels and thereby contributes to the energetic performance of cardiac muscle [35]. In short, the connection between these pathways indicated that cardiac muscle contraction, calcium signaling pathways and energy metabolism were cooperated with each other. Similarly, the enriched GO terms were predominantly associated with contractile muscle fibers and mitochondrial activity, further supporting a link between cardiac muscle structural integrity and energy metabolism.

Most genes enriched in pathways exhibited a high expression level. In detail, the cardiac muscle structures pathway is associated with the structural framework of cardiomyocytes, which consists of thin and thick filaments. The thin filaments are composed of troponins, which are encoded by three genes (*TNNT2*, *TNNI3*, and *TNNC1*), along with actins encoded by *ACTA1* (actin alpha 1) and *ACTC1* (actin alpha cardiac muscle 1) [36]. These thin filaments interact with thick filaments, which are primarily composed of myosin proteins (MYL2 (myosin light polypeptide 2), MYL3 (myosin light chain 3), MYH7 (myosin heavy chain 7), and MYBPC3), to regulate cardiac muscle contraction and relaxation [37,38,39]. During this process, TNNC1 serves as a calcium-sensitive unit to bind with Ca^2+^ [40], TNNI3 functions as an inhibitory subunit controlling muscle relaxation [41], and TNNT2 anchors the troponin complex to actin filaments [42]. Titin-cap (TCAP) is essential for sarcomere assembly, serving as a scaffold protein that anchors myofibrils and other muscle cells [43]. Among these genes, the expression levels of *TNNC1*, *TNNI3*, and *ACTC1* genes were higher in heart tissue than in arrhythmia patients [44]. Moreover, differential expressions of *MYBPC3*, *ACTC1*, *TCAP*, *TNNI3*, *MYL3*, and *MYH7* were also detected between PCV2-infected and healthy porcine cardiac tissues [45]. In this study, *TNNI3*, *TNNC1*, *ACTA1* and *ACTC1*, *MYH7* and *TCAP* were DEGs in at least two growing stages and also exhibited high expression levels, while *MYL3* and *MYBPC3* showed similarly high expression, suggesting these genes were essential for the maintenance of cardiac cell structure and functions. Furthermore, the changes in *COX4I1* and *ATP5MC1* expressions coincided with alterations in ATP levels [46,47]. The high expressions of the two genes in heart tissue further support a close association between cardiac muscle contraction and energy metabolism.

Notably, DEGs (*TNNI3*, *TNNC1*, *MYH7*, *ACTA1*, and *TCAP*) with high expression levels were gradually increased during the growing period, suggesting the functions of encoding proteins of thin and thick filaments are enhancing with age, whereas *MYL7* was the highest at the 3 days old age, and then began presenting a downward trend. *MYL7* is related to cardiac contraction by binding with Ca^2+^, and is a critical factor for cardiac development during the embryonic stage [48], indicating *MYL7* expression is most strongly associated with cardiac development before birth and at the neonatal stage. The upregulation of *GAPDH* pointed to heightened glycolytic energy production, while the downregulation of *ATP5MC1* and *COX4I1* suggested a role for these genes in oxidative phosphorylation before birth and at the neonatal stage.

### 4.3. Limitations of This Study

Among the 236 candidate genes identified, four cardiac genes (*COL3A1*, *TNNC1*, *ATM*, and *BMPR2*) are particularly worthy due to their evolutionary conservation between pigs and humans, and their established roles in cardiac physiology. While this study provides a genome-scale resource of selection signatures and variants for cardiac-related genes, the functional significances of these genes remain to be experimentally identified. These four conserved cardiac genes are predicted to be prioritized in future studies, including protein-level validation and transcriptional verification in larger grouping, to elucidate their contribution to cardiac functions in WZS pigs. Furthermore, the future research would be to conduct a genome-wide association study (GWAS) using the phenotypic traits of WZS pigs, which would allow assessment of the interaction between the cardiac-related genes identified in this study and quantitative trait loci (QTLs) of WZS pigs, and could enable more precise selection strategies in subsequent breeding programs. In addition, the RNA-seq analysis in this study was based on three biological replicates per time point (15 animals in total). Although this sample size is consistent with commonly adopted statistic numbers in the field and differentially expressed genes (DEGs) analysis employed stringent thresholds (|log_2_^FC^| ≥ 1, *p* ≤ 0.05), the statistical power of such a design is inherently limited. Therefore, the expression patterns of DEGs reported here should be interpreted with appropriate caution. Independent validation using RT-qPCR on an expanded sample size would be a valuable approach to confirm the key DEGs in future studies.

## 5. Conclusions

Between-breed analysis confirmed that WZS pigs presented higher nucleotide diversity than that of the other four pig breeds. A total of 236 genes were associated with cardiac contraction and relaxation, including 67 genes from the top 20 KEGG pathways, 192 genes from the top GO terms, 52 genes with high expression in the heart during five growing periods, and 44 top expression-lever DEGs. The functional enrichment pathways contained cytoskeleton in muscle cells, cardiac muscle contraction, gluconeogenesis and calcium signaling, which are cooperated together to support cardiomyocyte activities. DEGs with high expression are important to the cardiomyocyte structure and energy metabolism. The cardiac structure functions of thin and thick filament-encoding DEGs (*TNNI3*, *TNNC1*, *MYH7*, *ACTA1*, and *TCAP*) might be enhanced with increasing age, while *MYL7* expression was most strongly associated with cardiac development before birth and at the neonatal stage. *GAPDH*-related glycolysis heightened with increasing age, whereas *ATP5MC1* and *COX4I1* were most strongly linked with energy production before birth and at the neonatal stage. This study offers a list of cardiac-related genes for breeding and provides a fundamental basis for the development of porcine cardiac models for human disease research.

## Data Availability

The RNA-sequencing data were deposited at the National Center for Biotechnology Information (NCBI) Datasets under BioProject accession PRJNA1238565 (https://dataview.ncbi.nlm.nih.gov/object/PRJNA1238565?reviewer=pcnpth41btfqfklj6o1iealirf, accessed on 20 July 2026).

## Supplementary Materials

The following supporting information can be downloaded at: https://www.mdpi.com/article/10.3390/biology15141214/s1, Figure S1: the provinces where the samples were collected; Figure S2: the workflow of the cardiac-related genes selection; Figure S3: the proportion of variations in WZS pigs; Figure S4: the distribution of Tajima’s D value on WZS genomics; Figure S5: the relative divergencies of five pig breeds; Figure S6. the top 20 GO enrichment terms of the 192 genes; Table S1: the summary of whole-genome resequencing data from five breeds; Table S2: the nucleotide diversity of five pig breeds; Table S3: the genetic differences in five pig breeds; Table S4: FROH value of five pig breeds; Table S5: one-way ANOVA for FROH analysis; Table S6: the genes in the region of top 5% FST and top 5% π ratio (Duroc vs. WZS pigs); Table S7: the genes in the region of top 5% FST and top 5% π ratio (LW vs. WZS pigs); Table S8: the genes in the region of top 5% FST and top 5% π ratio (TC vs. WZS pigs); Table S9: the genes in the region of top 5% FST and top 5% π ratio (YX vs. WZS pigs); Table S10: the differences in five pig breeds; Table S11: high nucleotide diversity regions of WZS pigs; Table S12: high nucleotide diversity analysis of five pig breeds; Table S13: the TPM of 1095 genes in heart tissue from five growing periods; Table S14: the top 20 KEGG enrichment pathways of 67 genes in heart tissue of WZS pigs; Table S15: GO enrichment of 1095 genes in WZS pigs; Table S16: the conserved four genes in heart between pigs and human; Table S17: the RNA expression of the intersection of genes in the top 5% FST and π ratio (the other four pigs vs. WZS pigs and WZS pigs vs. the other four pigs); Table S18: the 44 differentially expressed genes from five growing periods in heart tissue of WZS pigs; Table S19: the mean and SD value of the top 12 expression-level DEGs; Table S20: RNA expression of selected 236 genes from five growing stages in heart tissue of WZS pigs.

## Abbreviations

The following abbreviations are used in this manuscript:

WZS pigs	Wuzhishan pigs
TC pigs	Tunchang pigs
YX pigs	Yuxi pigs
LW pigs	Large White pigs
BWA	Burrows–Wheeler Aligner
LD	Linkage disequilibrium
PSMC	Pairwise Sequentially Markovian Coalescent
NJ	Neighbor-joining
PCA	Principal component analysis
TNNC1	Troponin C1
TNNI3	Troponin I3
TNNT2	Troponin T2
ACTA1	Actin alpha 1
ACTC1	Actin alpha cardiac muscle 1
MYL2	Myosin light polypeptide 2
MYL3	Myosin light chain 3
MYH7	Myosin heavy chain 7
MYBPC3	Myosin binding protein C3
ATP5MC1	ATP synthase membrane subunit c locus 1
TCAP	Titin-cap
COX4I1	Cytochrome c oxidase subunit 4 isoform 1
COL3A1	Collagen type III alpha 1 chain
NCKAP1	NCK-associated protein 1
CALCRL	Calcitonin receptor-like receptor
BMPR2	Bone morphogenetic protein receptor type 2
