# Supplementary material for: Integrated Whole-Genome Resequencing and Transcriptomic Analyses to Reveal Breed-Specific Selection Signatures of Cardiac-Related Genes in Wuzhishan Pigs
Source: Biology (Basel). 2026 Jul 22;15(14):1214. doi: 10.3390/biology15141214 (PMC13405548; doi:10.3390/biology15141214)
Supplement: Supplementary file 1 [file biology-15-01214-s001.zip › Supplementary Figures revised-biology-4328068.pdf]

Supplementary figures

Figure S1. The provinces where the samples were collected
Figure S2. The workflow of the cardiac-related genes selection
Figure S3. The proportion of variations in WZS pigs
Figure S4. The distribution of Tajima's D value on WZS genomics
Figure S5. The relative divergencies of five pig breeds
Figure S6. The top 20 GO enrichment terms of the 192 genes

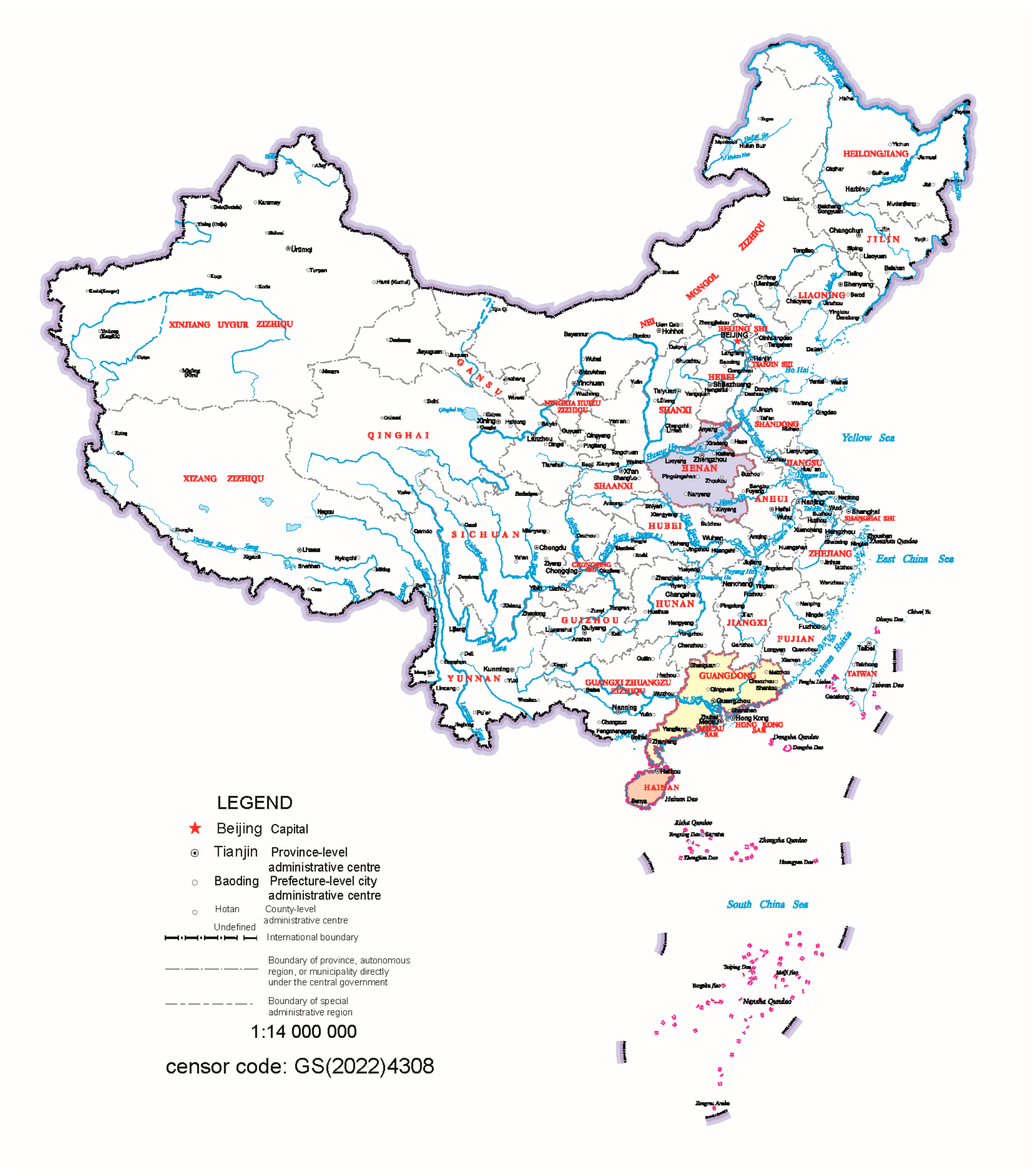

Figure S1. The provinces where the samples were collected

Three provinces with different colors were marked on the map. Chengmai City and Tunchang City, Hainan Province, China (Orange block with red circles): ears of 30 Wuzhishan (WZS) pigs and 30 Tunchang (TC) pigs were collected; Lushi City, western Henan Province, China (purple block with red circles): ears of 29 Yuxi Black (YX) pigs were sampled; Yunfu City, Guangdong Province, China (yellow block with red circles): ears of 30 Large White (LW) pigs and 28 Duroc pigs were obtained.

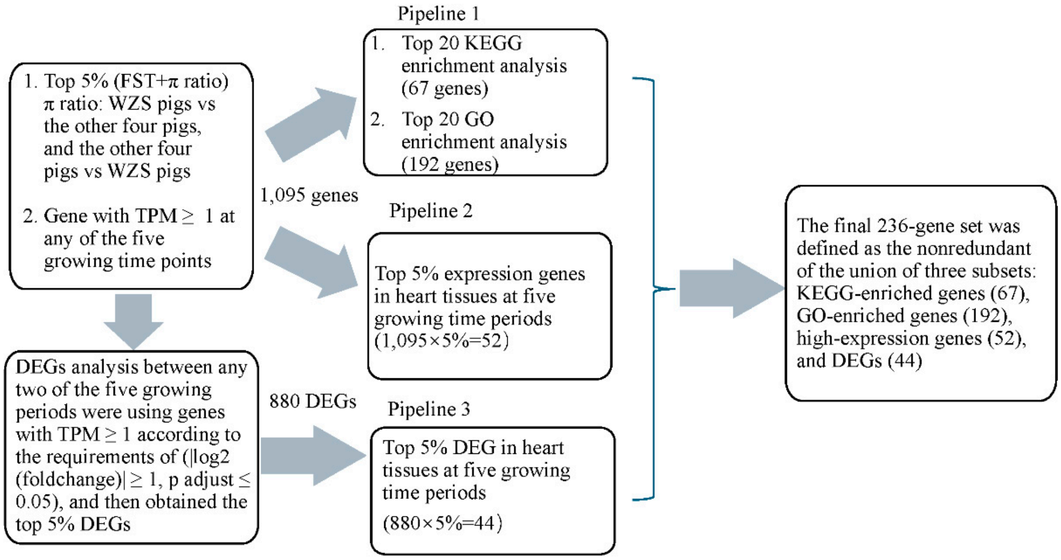

Figure S2. The workflow of the cardiac-related genes selection

The top left corner showed the initial gene set (1,095) were obtained from the top 5% FST and π ratio regions (π ratio: WZS pigs vs the other four pigs, and the other four pigs vs WZS pigs) with TPM ≥ 1, and the initial gene set was used to perform pipelines 1 and 2, yielding KEGG enriched genes (67), GO-enriched genes (129), and high-expression genes (52). Pipeline 3 was conducted based on the total DEGs, and obtained 44 DEGs. Finally, the union genes of the three pipelines were removed duplicate, and obtained a unique 236 genes.

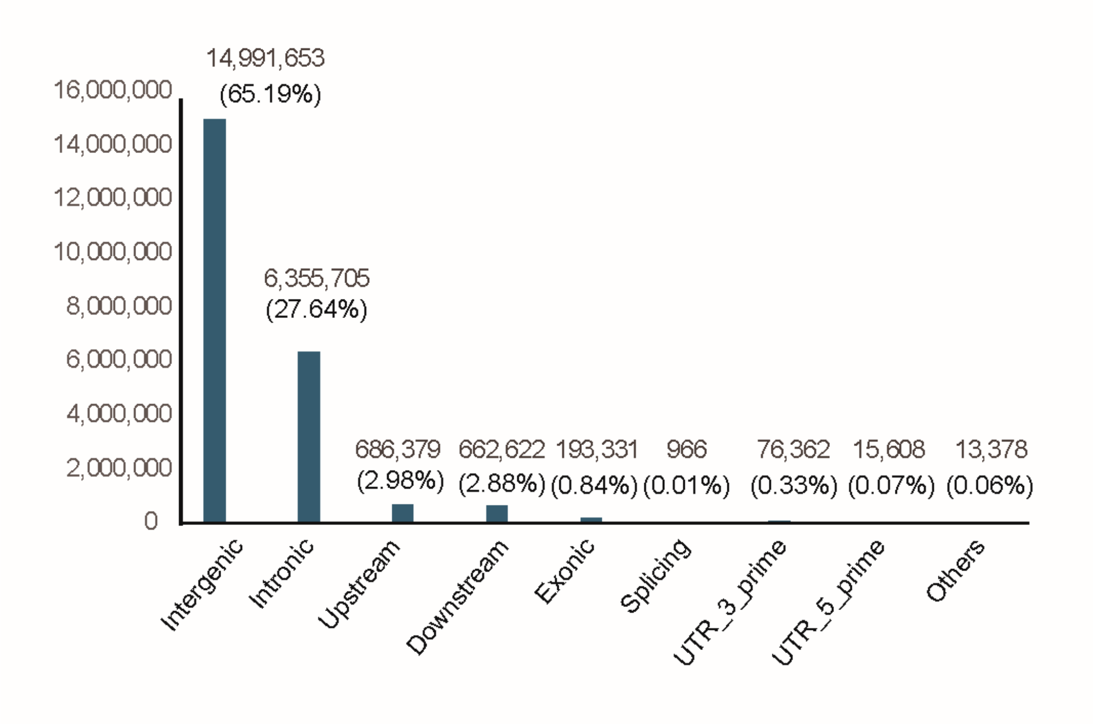

Figure S3 The proportion of variations in WZS pigs

The x-axis represents the variation types, and the y-axis represents the number of each variation. Each bar was marked with absolute values and percentages for a variation.

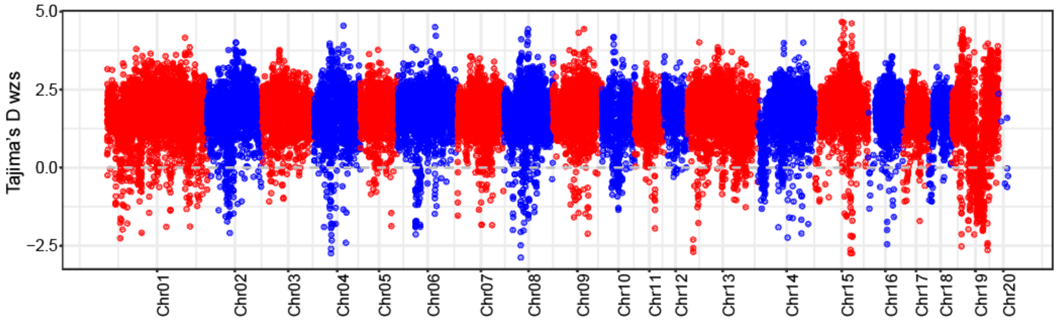

Figure S4. The distribution of Tajima's D value on WZS genomics.

Tajima's D was calculated using VCFtools with a sliding window 100 Kb.

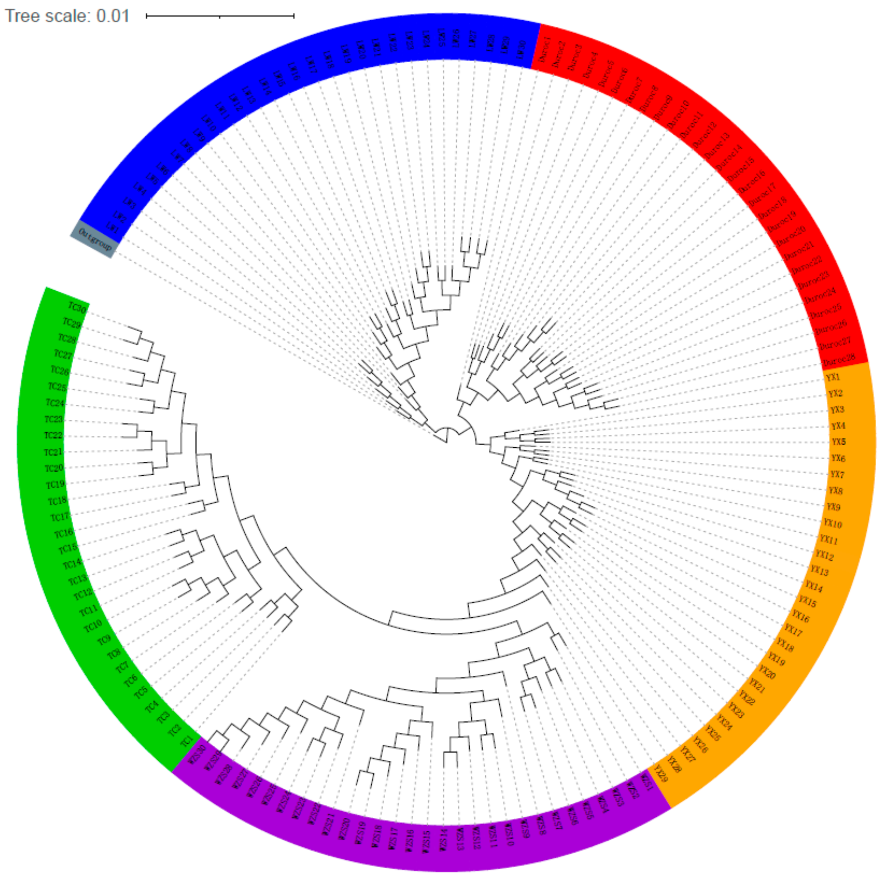

Figure S5. The relative divergencies of five pig breeds

Divergence times among five pig breeds were estimated using BEAST2, the outgroup was one cattle sample SRX23774943 in PRJNA658727, and the fossil time of Sus scrofa is 62 Mya (http://www.timetree.org/). The blue color, red color, orange color, purple color and green color exhibited individuals of 30 LW pigs, 28 Duroc pigs, 29 YX pigs, 30 WZS pigs, and 30 TC pigs.

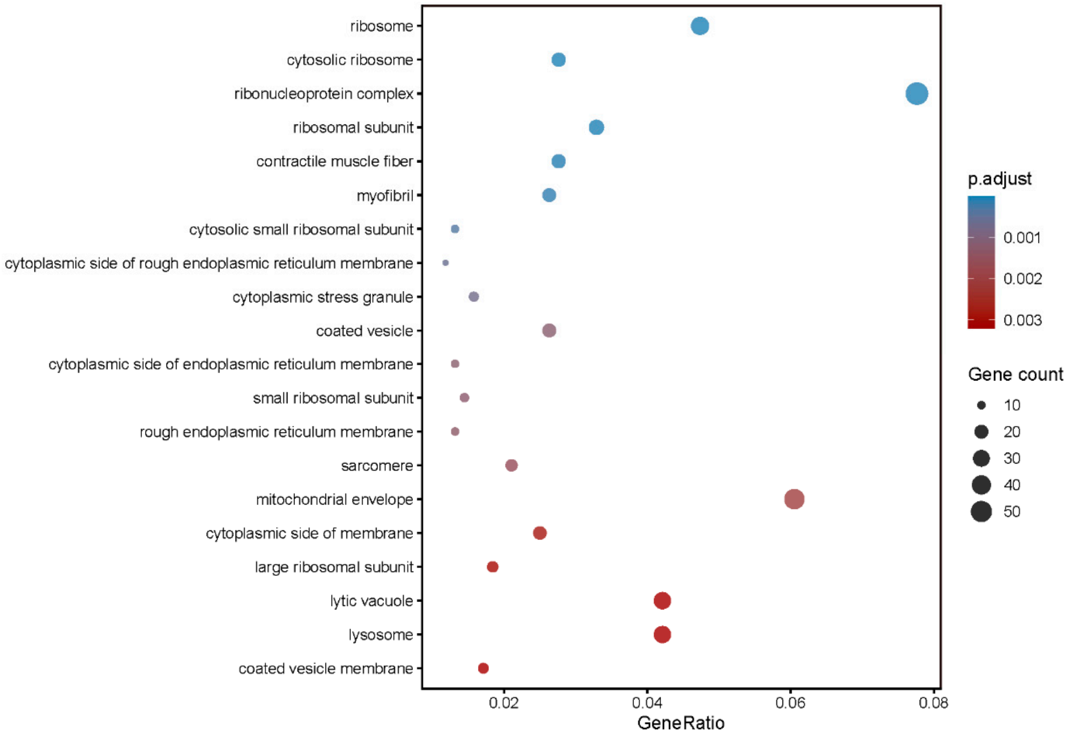

Figure S6. The top 20 GO enrichment terms of the 192 genes

The x-axis (gene ratio) represents the number of candidate genes annotated to a term divided by the total number of genes in that pathway. Dot size indicates the number of enriched genes; dot color indicates the adjusted P-value (-log10 transformed).
